# Multi-Nozzles 3D Bioprinting Collagen/Thermoplastic Elasto-Mer Scaffold with Interconnect Pores

**DOI:** 10.3390/mi16040429

**Published:** 2025-04-02

**Authors:** Kuo Yao, Kai Guo, Heran Wang, Xiongfei Zheng

**Affiliations:** 1 State Key Laboratory of Robotics, Shenyang Institute of Automation, Chinese Academy of Sciences, Shenyang 110016, China; yaokuo@sia.cn (K.Y.); guokai@sia.cn (K.G.); wangheran@sia.cn (H.W.); 2 University of Chinese Academy of Sciences, Beijing 100049, China

**Keywords:** collagen, hybrid scaffold, multimaterial printing, interconnect pores

## Abstract

Scaffolds play a crucial role in tissue engineering as regenerative templates. Fabricating scaffolds with good biocompatibility and appropriate mechanical properties remains a major challenge in this field. This study proposes a method for preparing multi-material scaffolds, enabling the 3D printing of collagen and thermoplastic elastomers at room temperature. Addressing the previous challenges such as the poor printability of pure collagen and the difficulty of maintaining structural integrity during multilayer printing, this research improved the printability of collagen by optimizing its concentration and pH value and completed the large-span printing of thermoplastic elastomer using a precise temperature-control system. The developed hybrid scaffold has an interconnected porous structure, which can support the adhesion and proliferation of fibroblasts. The scaffolds were further treated with different post-treatment methods, and it was proven that the neutralized and cross-linked collagen scaffold, which has both nano-fibers and a certain rigidity, can better support the osteogenic differentiation of bone marrow mesenchymal stem cells (BMSCs). The research results show that the collagen thermoplastic elastomer hybrid scaffold has significant clinical application potential in soft tissue and hard tissue regeneration, providing a versatile solution to meet the diverse needs of tissue engineering.

## 1. Introduction

Scaffolds have been a key element in tissue engineering and regenerative medicine as regenerative templates from the very beginning. Scaffold-based tissue engineering strategies offer a rapid opportunity for clinical transplantation of tissue engineering under the current medical device approval policy. However, after 40 years of tissue engineering research, there are still great technical barriers to the clinical translation of the scaffold concept, except for some simple tissue regeneration [1]. As an ideal scaffold for bioartificial tissue regeneration, the scaffold should simulate the geometric structure and biophysical and biochemical properties of the extracellular matrix (ECM) in vivo and have complex physical and chemical structural information [2]. Hollister summarized biomanufacturing and proposed the 4F principles for scaffold design and manufacturing: form, fixation, function, and formation [3]. Due to the requirement for multiple interconnected properties, the manufacturing of scaffolds that meet all properties has not yet been successful.

Biological materials for tissue engineering scaffolds can generally be divided into two major categories: natural polymers and synthetic polymers [4]. Natural biopolymers have good cell compatibility but poor mechanical properties. Collagen, the main structural protein of the extracellular matrix (ECM) in vertebrate connective tissue, has good biocompatibility and is the preferred material for current tissue engineering scaffolds, with type I collagen being the most commonly used. Many collagen-based products have been applied in clinical surgery. In addition to simple collagen medical products, obtaining collagen scaffolds with controllable structures and excellent mechanical properties through bioprinting has always been considered a promising method for clinical application. However, it is difficult to meet the mechanical requirements of scaffolds using only collagen materials, so the composite manufacturing of collagen and synthetic polymers is considered. The printing technology for single synthetic polymers has been mature, but the 3D printing of collagen and the mixed printing of collagen and synthetic polymers have not been well achieved yet.

The printing of collagen–synthetic polymer hybrid scaffolds mainly faces two problems. First, pure collagen has poor printability. Although methods such as low-temperature printing [5], embedded printing [6,7], or doping with thickeners (such as alginate [8], hyaluronic acid [9], etc.) can improve it, these methods all have some problems, such as nozzle clogging, filament bonding issues, and reduced biocompatibility. A Lee [10] used the embedded printing method and successfully printed collagen heart scaffolds with lower concentrations in a gelatin microsphere support bath. The support bath can melt at 37 degrees, ensuring the structural integrity of the scaffold. However, this method is not suitable for multi-material printing, especially the printing of collagen and synthetic polymers together, and it is difficult to complete the formation of hybrid scaffolds. Another problem is that it is difficult to meet the preparation and printing conditions for both collagen and synthetic polymers (such as temperature, cross-linking conditions, etc.) at the same time. The common method is to print porous polymer scaffolds first and then cast collagen to form the scaffold [11]. However, these scaffolds often fail to meet the optimal pore conditions, which is not conducive to cell proliferation and differentiation. YoungWon Koo [12] used temperature-controlled technology to prepare collagen/PCL hybrid scaffolds. However, this method could only retain the formation of interpenetrating pores in PCL single-material printing. When preparing hybrid scaffolds, collagen/PCL would collapse, losing the characteristic of lateral pores.

In the clinical application of tissue engineering scaffolds, various factors affecting the scaffold’s functionality should be considered comprehensively. This study developed a new printing technology. Through rheological analysis, we identified the collagen preparation conditions suitable for room-temperature printing in the air. Using a precise temperature-control system, we can complete the large-span printing of PCL and prepare collagen-synthetic polymer hybrid scaffolds with interconnected pores. Moreover, by adjusting the ratio of collagen and PCL, the mechanical properties of the hybrid scaffold can be regulated to meet different tissue regeneration needs. Additionally, we used fibroblasts and bone marrow mesenchymal stem cells as experimental cells for soft tissue and hard tissue regeneration, respectively, to assess the biocompatibility and mechanical properties of the hybrid scaffolds. Based on these results, the proposed hybrid scaffold printing method holds great clinical application value in tissue regeneration.

## 2. Materials and Methods

### 2.1. Preparation and Rheology of Bioinks

Type I collagen molecules were extracted from bovine tendons using a classical procedure. The brief process is as follows: Fresh tendons were cut into small pieces and soaked in a 0.8% sodium carbonate solution for 12 h to remove fat. After being washed multiple times with deionized water (dH_2_O), the tendon pieces were dissolved in a solution containing 0.2 M acetic acid and 0.25% (*w*/*v*) pepsin and reacted under stirring for 84 h. The crude solution was centrifuged, and the supernatant was selectively precipitated with 1 M NaCl. The precipitated type I collagen was dissolved in 0.2 M acetic acid, desalted by dialysis against 0.2 M acetic acid, and adjusted to the final concentration by dialysis against polyethylene glycol. During dialysis, dH_2_Oshould be changed every 24 h, and the pH of dH_2_Oshould be monitored with a pH meter. Dialysis was terminated when the pH reached the desired value. The solution then was lyophilized for 1 week to generate a white porous foam and stored at 4 °C. A predetermined mass of collagen foam was weighed and dissolved in phosphate buffer saline (PBS) to prepare collagen solutions at varying concentrations before printing.

Medical-grade polycaprolactone (PCL) with an average molecular weight of 15,000 and an intrinsic viscosity ranging from 1.75 to 2.25 dL/g was purchased from ShenZhen Polymtek Co., Ltd. in Shenzhen, China. PCL pellets were introduced into a planetary ball mill (XQM-2A, purchased from Shanghai, China) and ground for 1 h at a rotational speed of 300 rpm. Following the grinding process, the PCL powder was transferred to a filament extruder (Wellzoom Desktop Filament Extruder B-pro, purchased from Shanghai, China), where it was extruded into PCL rods via a screw mechanism. The PCL rods can be stored at room temperature and, prior to printing, were loaded into the printing chamber and heated to a molten state.

The rheological properties of the collagen solution were measured using an Anton Paar MCR 501 rheometer (Anton Paar GmbH, Ostfildern, Germany). The PP25 parallel plate measuring system was employed, with a gap setting of 0.3 mm and a temperature of 25 °C.

### 2.2. Preparation of the Hybrid Scaffold

**Figure 1:**
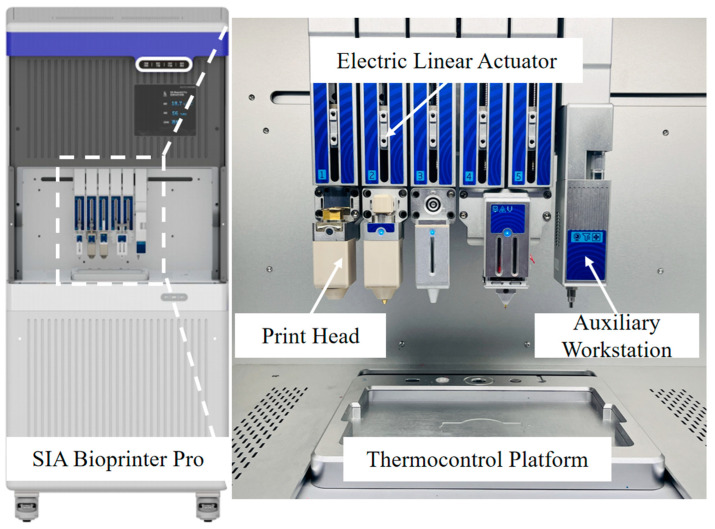
Structural composition of “SIA Bioprinter Pro”.

The scaffold was accomplished using a bioprinter (SIA Bioprinter Pro, developed by our research team, Figure 1). In previous work, we have utilized this printer for the bioprinting of various elastomers and hydrogels [13,14,15,16]. High-precision extrusion-based printing of collagen and PCL was achieved using an electric linear actuator. The temperature control accuracy of the nozzle and the working platform was within 1 degree. During the printing process, stainless steel needles with diameters of 150 μm, 200 μm, 500 μm, and 600 μm were used. The printing paths for multiple nozzles were generated using Matlab(R2023b) software. To prevent collagen from drying, the printing environment was maintained at 90% relative humidity (RH). After printing, different post-treatment methods were used. For the freeze-drying group, the scaffold was cooled to −80 °C and then freeze-dried using a freeze-dryer (Alpha 1–2 LD plus, Christ, Osterode, Germany).

### 2.3. Overall Preparation Process of the Hybrid Scaffold

**Figure 2:**
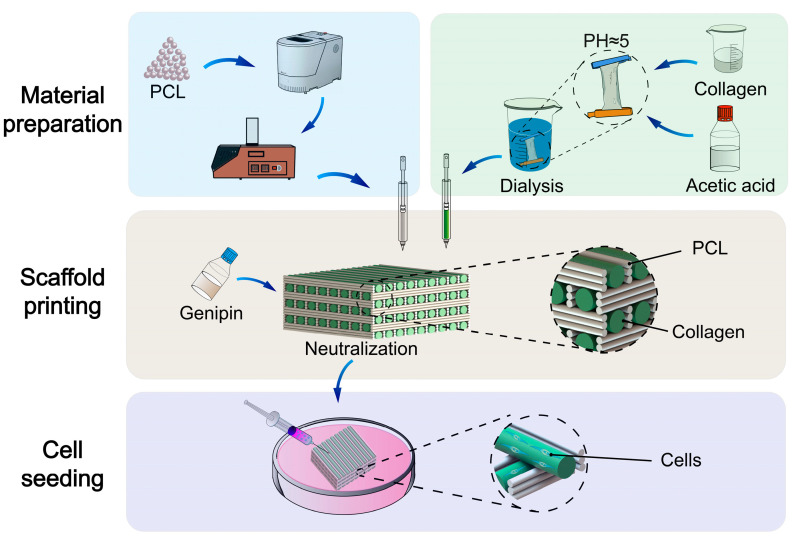
Overall preparation process of the hybrid scaffold.

The overall fabrication process of the hybrid scaffold is shown in Figure 2. Its three basic fabrication stages can be summarized as follows:

- Material preparation: Two types of bioinks were prepared according to the procedure described in Section 2.1. After preparation, the bioinks were transferred into the printing tubes for scaffold printing.
- Scaffold printing: The hybrid scaffold was manufactured at room temperature through a precise temperature-control system. The printing parameters were optimized to maintain the pores between the polymer and collagen fibers, thereby promoting subsequent cell proliferation. After printing, the scaffold was post-treated according to the method described in Section 2.5, forming a collagen fiber structure that promotes cell proliferation.
- Cell seeding: The cross-linked scaffold was placed in the culture medium and incubated for 24 h. Subsequently, different types of cells were seeded onto the scaffold and continued to culture. These cells attached to the collagen fibers and underwent proliferation and development.

### 2.4. Physicochemical Tests of Scaffold

#### 2.4.1. Macroscopic and Microscopic Structures

The macroscopic and microscopic structures of the scaffold were observed using a scanning electron microscope (SEM). First, the scaffold was treated with genipin and then freeze-dried. A layer of ultra-thin Aurum/Platinum (Au/Pt) was applied to the freeze-dried scaffold for sample preparation and SEM imaging.

#### 2.4.2. Mechanical Testing

Collagen/PCL scaffolds with different ratios were fabricated into a uniform size of 15 × 15 × 5 mm (L × W ×H). Compressive tests were performed using a mechanical analyzer (CT3 texture analyzer, 100 g/1500 g, AMETEK Brookfield, Middleboro, MA, USA).

#### 2.4.3. Sodium Dodecyl Sulfate Polyacrylamide Gel Electrophoresis (SDS–PAGE)

SDS-PAGE was performed using 6% (*w*/*v*) normal collagen prepared in the laboratory and 6% (*w*/*v*) collagen from the hybrid scaffold that had been in contact with PCL. The collagen protein was dissolved in a 0.02 M sodium phosphate buffer (pH 7.2) containing 1% (*w*/*v*) SDS and 3.5 M urea to obtain a final concentration of 2 mg/mL. It was then mixed with an equal volume of sample buffer (0.5 M Tris-HCl, pH 6.8, containing 4% (*w*/*v*) SDS and 20% (*v*/*v*) glycerol). Subsequently, 20 μL of the sample (20 μg of protein) was loaded into each well. High-molecular-weight markers were used to estimate the molecular weight of the bands. After gel electrophoresis, the gel was stained with 0.1% (*w*/*v*) Coomassie Brilliant Blue R–250 in a solution of 50% (*v*/*v*) methanol and 6.8% (*v*/*v*) glacial acetic acid for 5 h. It was then destained with a solution of 7.5% (*v*/*v*) glacial acetic acid and 5% (*v*/*v*) methanol for about 9 h, with the solution changed every 3 h.

### 2.5. Post-Treatment Methods of the Scaffold

Three methods were used to treat collagen/polycaprolactone hybrid scaffolds. In the first method, after printing, the collagen scaffolds were frozen at −80 °C and then freeze-dried. They were incubated in a 1% (*w*/*v*) genipin ethanol solution for 12 h to cross-link the collagen. After washing the scaffolds three times with ethanol and distilled water, they were blocked with a 1% (*w*/*v*) glycine solution to neutralize residual cross-linking agents. This group was named the freeze-dried and cross-linked group.

In the second method, 10 × PBS was used to induce fiber formation. The collagen scaffolds were soaked in 10 × PBS at 37 °C for 1 h. Then, the samples were taken out from the 10 × PBS solution and washed three times in 1 × PBS. The samples were stored in 1 × PBS until needed and named the neutralized group.

The third method involved immersing the 10 × PBS-treated scaffold material in a 1% (*w*/*v*) genipin solution for 12 h to stabilize the fiber structure and prolong degradation time. After washing the scaffolds three times with PBS, they were blocked with a 1% (*w*/*v*) glycine solution to neutralize residual cross-linking agents. This group was named the neutralized and cross-linked group.

### 2.6. Cell Culture Experiments

#### 2.6.1. Rabbit Bone Mesenchymal Stem Cells (BMSCs)

Rabbit BMSCs were purchased from Cyagen Biosciences, Inc(Suzhou, China). The cells were expanded at 37 °C and 5% CO_2_ in Dulbecco’s modified eagle’s medium low glucose (DMEM; Gibco, Life Technologies, Shanghai, China) supplemented with 10% fetal calf serum (Gibco, Life Technologies), 100 U mL^−1^ penicillin, and 100 μg · mL^−1^ streptomycin (Gibco, Life Technologies). Cells in passage 6 were used for the experiments.

#### 2.6.2. Human Dermal Fibroblasts (FBs)

Human FB cells were purchased from ScienCell Research Laboratories, Inc. (Carlsbad, CA, USA). The cells were expanded at 37 °C and 5% CO^2^ in Dulbecco’s modified eagle’s medium high glucose (DMEM; Gibco, Life Technologies) supplemented with 10% fetal calf serum (Gibco, Life Technologies), 100 U mL^−1^ penicillin, and 100 μg · mL^−1^ streptomycin (Gibco, Life Technologies). Cells in passage 6 were used for the experiments.

### 2.7. Cell Seeding in Collagen Scaffolds

The produced scaffolds were incubated in cell culture medium for 24 h and thereafter seeded at a certain amount of cells in culture medium in 24-well cell culture plate (Corning 3473 Ultra-Low Attachment, Corning, NY, USA). Osteogenic differentiation of BMSC was induced by addition of 100 nM dexamethasone, 3.5 mM β-glycerophosphate, and 0.05 mM ascorbic acid 2-phosphate (all from Sigma-Aldrich, St. Louis, MO, USA) to the cell culture medium. Induction of osteogenic differentiation was started 7 days after seeding.

### 2.8. Analysis of Cell Adhesion, Proliferation

Cell proliferation on the scaffolds was evaluated at 1, 10, and 21 days post-seeding. To quantify the attached cells, the scaffolds were treated with 0.25% trypsin-EDTA solution at 37 °C for 10 min to detach the cells. Subsequently, the cell suspension was collected for counting. For qualitative assessment, the samples were incubated with Calcein-AM solution (Life Technologies, Carlsbad, CA, USA) and visualized using a fluorescence microscope (Leica DMI8, Wetzlar, Germany).

### 2.9. Analysis of BMSC Osteogenesis Differentiation in Hybrid Scaffold

#### 2.9.1. Alizarin Red S Stain

After washing twice with PBS, the samples were immersed in 4% (*w*/*v*) paraformaldehyde for 15 min and stained with 1% (*w*/*v*) alizarin red S (Sigma) for 20 min to observe under a light microscope.

#### 2.9.2. Von Kossa Stain

After washing twice with PBS, the samples were immersed in 4% (*w*/*v*) paraformaldehyde for 15 min and soaked in 5% (*w*/*v*) silver nitrate solution under UV light for 10 min. After soaking in sodium thiosulfate for 10 min, scaffolds were observed under a light microscope.

#### 2.9.3. Alkaline Phosphatase (ALP) Staining and Activity Assay

After induction for 21 days, the scaffolds were rinsed three times with PBS for ALP staining via a BCIP/NBT ALP color development kit (Beyotime, Shanghai, China). ALP assay kit (Beyotime, China) was used for quantitative detection of ALP activity. Each sample in 24-well plate was washed with PBS and incubated in 100 μL 1% (*w*/*v*) Triton X-100 in PBS for 30 min. A total of 100 μL of substrate solution was added to each well. After incubation at 37 °C for 30 min, the enzymatic reaction was stopped by addition of 1 M NaOH; pnitrophenolate (pNp) formation was quantified by absorbance measurement at 405 nm. The amount of pNp produced by each sample was calculated using ap-nitrophenol calibration line, and ALP activity (μmol pNp/30 min/one scaffold) was defined.

### 2.10. Real-Time Polymerase Chain Reaction (PCR)

The Exicycler^TM^ 96 real-time PCR detection system (BIONEER, Daejeon, Republic of Korea) was used to perform RT–PCR experiments. Ribonucleic acid (RNA) was extracted from the printed samples cultured in osteoblastic medium for 21 days. The expression levels of osteogenesis–related genes, including alkaline phosphatase (ALP), bone sialoprotein (BSP), osteocalcin (OCN), and collagen type I alpha 1 (COLLA1), were used to represent the fold changes in target gene expression.

### 2.11. Statistical Analysis

All data were presented as means ± standard deviations. Statistical significance was determined by analysis of variance with Tukey honest significant difference post hoc as * *p* < 0.05, ** *p* < 0.01, and *** *p* < 0.001.

## 3. Results

### 3.1. Printability of Collagen

**Figure 3:**
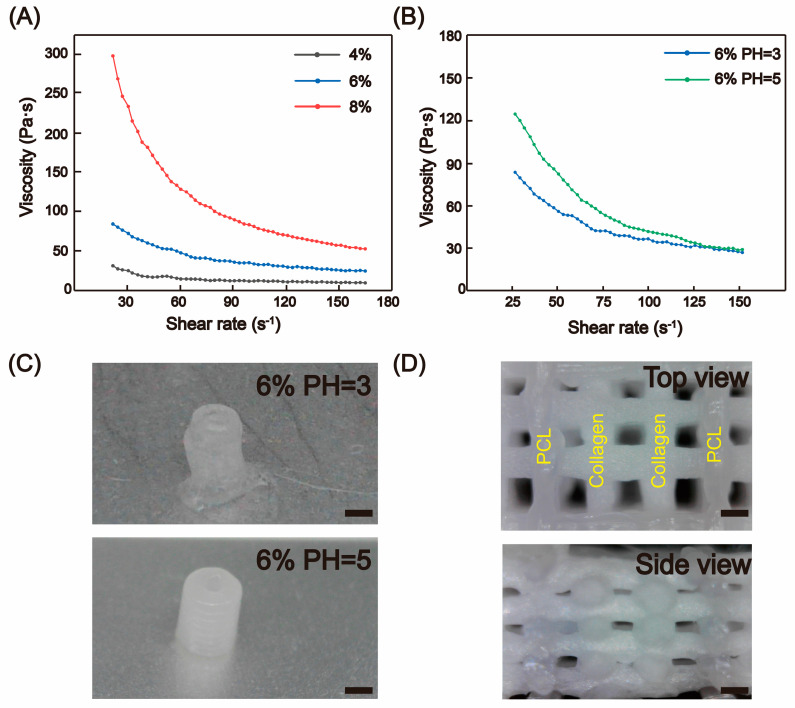
Rheological properties and printability of collagen. (**A**) Shear-thinning behavior of collagen at different concentrations (4% *w*/*v*, 6% *w*/*v*, 8% *w*/*v*) under pH = 3. (**B**) Shear-thinning behavior of 6% *w*/*v* collagen at pH = 3 and pH = 5. (**C**) Photograph of a collagen-printed cylinder, scale bar: 2 mm. (**D**) The hybrid scaffold printed with collagen and PCL, scale bar: 500 μm.

Shear-thinning is a critical property for evaluating the printability of bioinks [17]. In the case of collagen solutions, the entanglement of macromolecules, along with polar and hydrophobic interactions, contributes to their high viscosity. These non-covalent forces are readily disrupted by shear stress, leading to rapid rearrangement and the manifestation of shear-thinning and self-healing behaviors [18]. For scaffold printing, it is essential that collagen exhibits shape retention in a static state, transitions to a liquid-like state under shear stress, and rapidly reverts to a solid-like state upon the removal of shear forces. Concentration is a primary factor influencing rheological properties. When the collagen concentration is below 4%, the material demonstrates poor formability and is prone to collapse, making it unsuitable for printing tissue engineering scaffolds with specific pore size requirements. Therefore, we consider 4% to be the minimum concentration for printing porous scaffolds in air. Rheological testing of collagen at pH 3 across different concentrations (Figure 3A) revealed that the initial viscosities of 4%, 6%, and 8% collagen solutions were approximately 195 Pa∙s, 282 Pa∙s, and 583 Pa∙s, respectively. The viscosity of collagen increased with concentration, and all groups exhibited shear-thinning behavior, with the degree of shear-thinning enhancement correlating with higher concentrations. However, increasing the concentration is not without limits. At excessively high initial viscosities (e.g., 8%), collagen exhibits overly high viscosity at low shear rates, leading to nozzle clogging and significantly prolonged relaxation times, which complicates control and compromises printing precision. Thus, an appropriate initial viscosity and suitable shear-thinning capacity are crucial criteria for selecting collagen for printing. Based on experimental results, we determined that collagen with an initial viscosity ranging from 230 Pa∙s to 450 Pa∙s is suitable for scaffold printing at room temperature. Consequently, we preliminarily selected 6% collagen (282 Pa∙s) as the optimal concentration for printing.

pH is another important factor affecting the rheological properties of collagen. For type I collagen, within the pH range of 3–7, its viscosity generally increases as the pH decreases. However, different rheological properties were observed in dialyzed collagen. We noticed that for 6% collagen, the viscosity at pH 5 was higher than that at pH 3, and the viscosity decreased more rapidly with increasing shear rate, indicating enhanced shear-thinning behavior at pH 5 (Figure 3B). The reason might be that at this pH value, partial gelation occurred between collagen molecules during dialysis. Under high-concentration conditions, the distance between partially assembled collagen fibers was closer, making it easier for them to affect each other and form an aggregation effect, thereby increasing the viscosity.

We know that the dissolution of high-concentration collagen is difficult and has limited solubility. Therefore, by adjusting the pH during collagen dialysis, it is possible to meet the viscosity requirements for collagen printing at a relatively lower concentration and reduce the preparation time. We finally selected a collagen concentration of 6% and pH = 5 as the final preparation parameters. The selected collagen exhibited a viscosity of approximately 356 Pa∙s at room temperature, demonstrating excellent shear-thinning properties, which enabled superior printability under ambient conditions (Figure 3C).

### 3.2. Large-Span Printing of PCL

**Figure 4:**
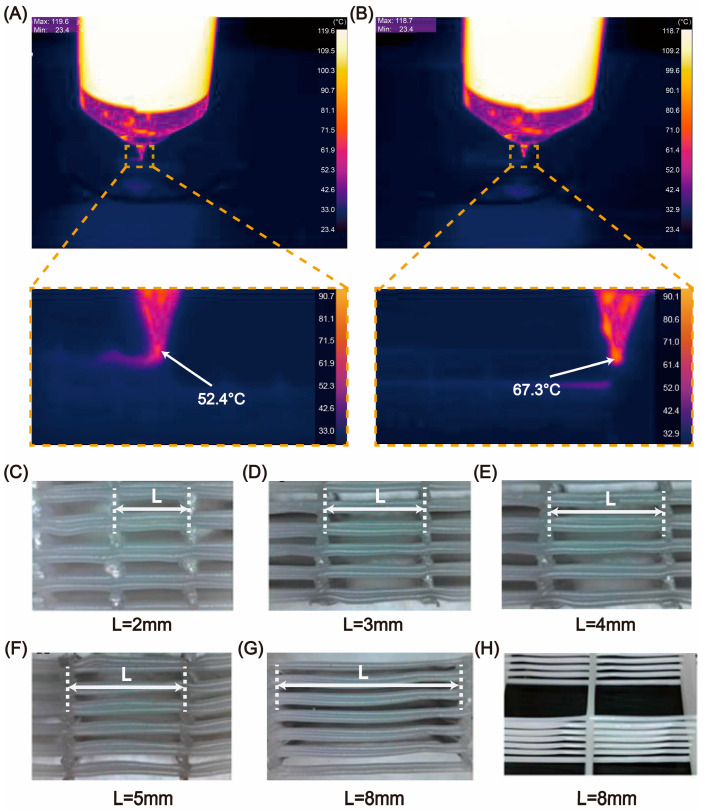
Large-span printing of PCL. (**A**,**B**) Thermal imaging of printing temperature when PCL is not prone to collapse. (**C**–**H**) Printing of pure PCL scaffolds at different spans (2 mm, 3 mm, 4 mm, 5 mm, and 8 mm).

The mechanical strength of the hybrid scaffold mainly depends on the bonded PCL network, so this study does not explore the bonding relationship between PCL and collagen. For different tissue regeneration, the ratio of PCL and collagen required in the scaffold will also change accordingly. When the scaffold requires a higher proportion of collagen, it needs PCL to have the ability of large-span printing (Figure 3D). We noticed that by controlling the appropriate printing temperature and speed, the PCL filament can be quickly solidified and not collapse after extrusion, thus realizing the goal of large-span printing. We carried out PCL printing experiments at multiple temperatures and finally confirmed that the printing effect of PCL was the best when the nozzle temperature was in the range of 52.4–67.3 °C (Figure 4A,B). Based on the precise temperature-control system, we successfully realized the printing of PCL scaffolds with different spans (Figure 4C–H). The PCL filaments all exhibited a good linear morphology without collapse.

### 3.3. Printing of the Hybrid Scaffold

**Figure 5:**
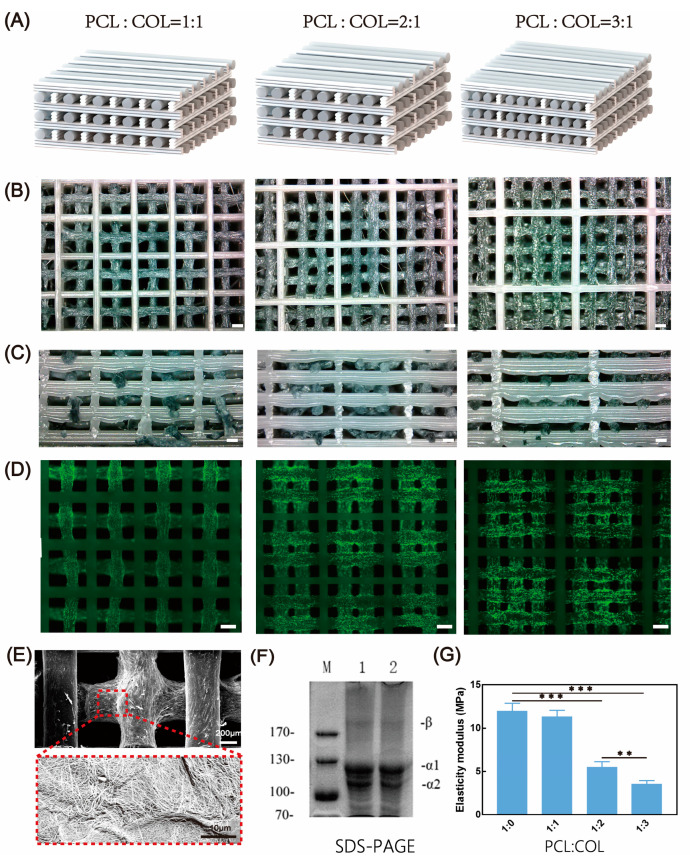
Design, printing, seeding, and mechanical strength testing of the hybrid scaffold. (**A**) Design of hybrid scaffolds with different collagen/PCL ratios (we defined the number of filaments visible in the XY plane as the design ratio). (**B**) Top view of the hybrid scaffold. (**C**) Side view of the hybrid scaffold. (**D**) Fluorescent images of the scaffold after cell seeding. (**E**) Nano-fiber structure formed in the hybrid scaffold after neutralization treatment. (**F**) SDS–PAGE results of collagen, where 1 is normal collagen and 2 is collagen that came into contact with PCL during the printing of the hybrid scaffold. (**G**) Mechanical properties of the hybrid scaffold under different ratios, N = 3; data are means ± SD. ** *p* < 0.01 and *** *p* < 0.001. Scale bars in (**B**–**D**): 500 μm.

During the printing of hybrid scaffolds, the high-precision printing of PCL is more controllable compared to collagen. To achieve optimal porosity while minimizing the impact of PCL’s high temperature on collagen within the same layer, we reduced the diameter of PCL filaments to one-third that of collagen filaments. Although PCL and collagen do not come into contact, slight contact between different layers is inevitable. To assess the impact of the instantaneous high temperature of PCL extrusion on collagen hydrogel, the SDS-PAGE method was used to detect the thermal degradation of collagen (Figure 5F). The results showed that the physical properties of collagen did not change, thus ensuring the bioactivity of collagen and its clinical application.

Taking advantage of the printability of collagen at room temperature and the large-span printing capability of PCL, we developed scaffold models with different composition ratios (Figure 5A) to achieve customized mechanical properties and cell seeding density for specific tissue requirements. Thanks to the excellent printing and forming performance of the ink, the designed lateral pores in the hybrid scaffold will not collapse while ensuring the original positive pores (Figure 5C). This interpenetrating porous structure is conducive to the full transportation of nutrients. The collagen content in the scaffold is directly related to its cell adhesion ability. For soft tissue applications that require high cell density and low mechanical strength, collagen-rich scaffolds are more suitable. In contrast, hard tissue regeneration may benefit more from hybrid scaffolds with lower collagen content. This compositional flexibility allows us to precisely adjust the performance of the scaffold to meet the diverse needs of tissue engineering.

In the context of bioprinting, scaffolds constructed from bioinks partially mimic the role of the extracellular matrix (ECM), and the stiffness of the ECM plays a critical role in regulating cell adhesion, proliferation, and differentiation. Generally, cells tend to spread more extensively and adopt a flattened morphology on stiffer substrates, whereas they remain rounded and exhibit limited spreading on softer substrates. Therefore, modulating the mechanical strength of scaffolds within a certain range is essential for cell and tissue culture. Through mechanical testing of scaffolds with varying compositions, we observed that the mechanical properties of the PCL–collagen (1:1) hybrid scaffold were comparable to those of pure PCL scaffolds. This indicates that the incorporation of collagen filaments did not significantly affect the bonding of PCL filaments during the printing process. When the proportion of PCL was reduced, the elastic modulus of the hybrid scaffolds decreased accordingly. This result is expected, as the mechanical properties of the hybrid scaffolds are primarily provided by PCL. Thus, the mechanical properties of the scaffolds can be modulated within a certain range by adjusting the proportion of PCL. The scaffolds developed in this study are primarily intended for connective tissues, with a focus on mechanical performance for implantation. Both PCL and collagen are biocompatible materials, and the interconnected pores of the scaffold meet the porosity requirements for cell growth. Under these conditions, the mechanical properties of the scaffold are designed to match those of the target regenerated tissue, thereby fulfilling the criteria for implantation [19,20]. In scaffolds where PCL serves as the elastomer, the elastic modulus of the hybrid scaffolds ranged from 4 to 12 Mpa (Figure 5G), which meets the conditions for osteogenic differentiation and supports the proliferation of mesenchymal stem cells (MSCs). Additionally, the printing methodology developed in this study is applicable to a variety of other materials (e.g., PLCL). In future work, we will explore the printing of hybrid scaffolds using other elastomers/hydrogels combined with collagen, aiming to fabricate scaffolds that meet the mechanical requirements for the differentiation and growth of other cell types.

### 3.4. Biological Evaluation

#### 3.4.1. Cell Seeding

**Figure 6:**
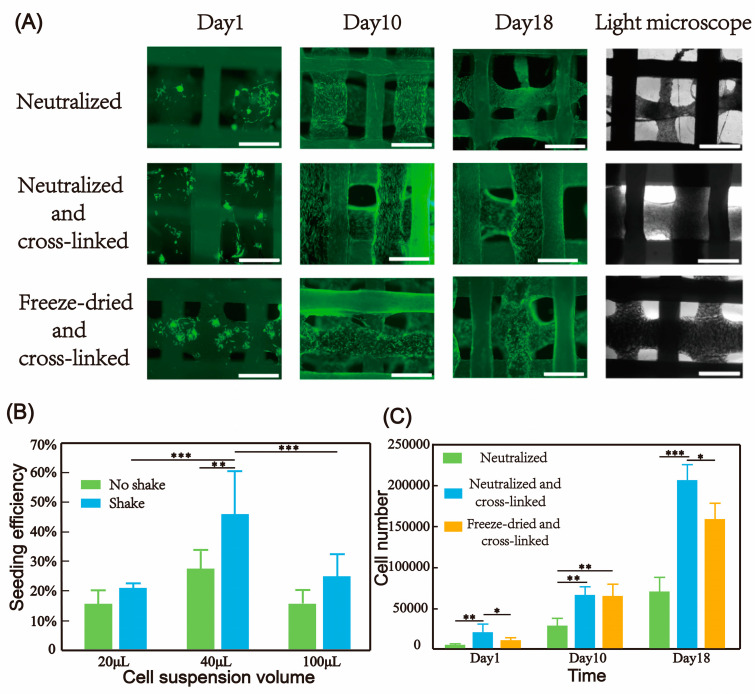
Culture of cells in the hybrid scaffold. (**A**) Fluorescent images and bright-field microscopic images of the hybrid scaffold after different post-treatment on days 1, 10, and 18 after cell seeding, scale bar: 500 μm. (**B**) The relationship between cell seeding methods and seeding efficiency, N = 3; data are means ± SD. ** *p* < 0.01 and *** *p* < 0.001. (**C**) The relationship of cell numbers in the hybrid scaffold after different post-treatments on days 1, 10, and 18, N = 3; data are means ± SD. * *p* < 0.05, ** *p* < 0.01, and *** *p* < 0.001.

Fibroblasts at different concentrations were seeded onto the scaffolds that had been freeze-dried and cross-linked. During the seeding process, some scaffolds underwent gentle agitation, with a cell density of 5 × 10^4^ cells per scaffold. The scaffolds measured 5 mm × 5 mm × 1 mm in size. Three hours later, the cells on the scaffolds were digested with trypsin and counted. The results indicated that when the cell concentration was 1.25 × 10^6^ (40 μL) and gentle agitation was performed, the cell seeding efficiency reached the highest level of approximately 46% (Figure 6B). The seeding efficiency of scaffolds with different collagen ratios was also evaluated. The findings revealed that for scaffolds of the same volume, the number of attached cells increased as the collagen content increased (Figure 5D). One day after seeding, the scaffolds were sectioned to examine the distribution of cells inside. Due to the porous nature of the scaffold, the cells were well distributed within the structure.

#### 3.4.2. Cultivation of Human Dermal Fibroblasts (FBs) in Hybrid Printing Scaffold

To meet the diverse strength requirements, as described in Section 2.4, scaffolds were prepared using multiple post-treatment methods, namely the neutralized group, the neutralized and cross-linked group, and the freeze-dried and cross-linked group. Cells on the surface of scaffolds in different groups exhibited typical spreading morphologies. Moreover, in the neutralized and neutralized and cross-linked groups, we could observe the arrangement of collagen fibers (Figure 5E), and cells would spread along the printing direction. On day 10, the cell numbers in all three groups were significantly higher than those on day 1 (Figure 6C), indicating that the toxic effects of genipin were minimal after glycine treatment. On day 18, cells in the neutralized and cross-linked group and the freeze-dried and cross-linked group continued to proliferate at a faster rate, while the cell number in the neutralized group remained consistent with that on day 10, which might be due to the degradation of collagen in the neutralized group. On day 18, the cell number in the neutralized and cross-linked group was higher than that in the freeze-dried and cross-linked group, indicating that the presence of collagen fibers was more conducive to cell proliferation.

#### 3.4.3. Cultivation of BMSC in Hybrid Printing Scaffold

**Figure 7:**
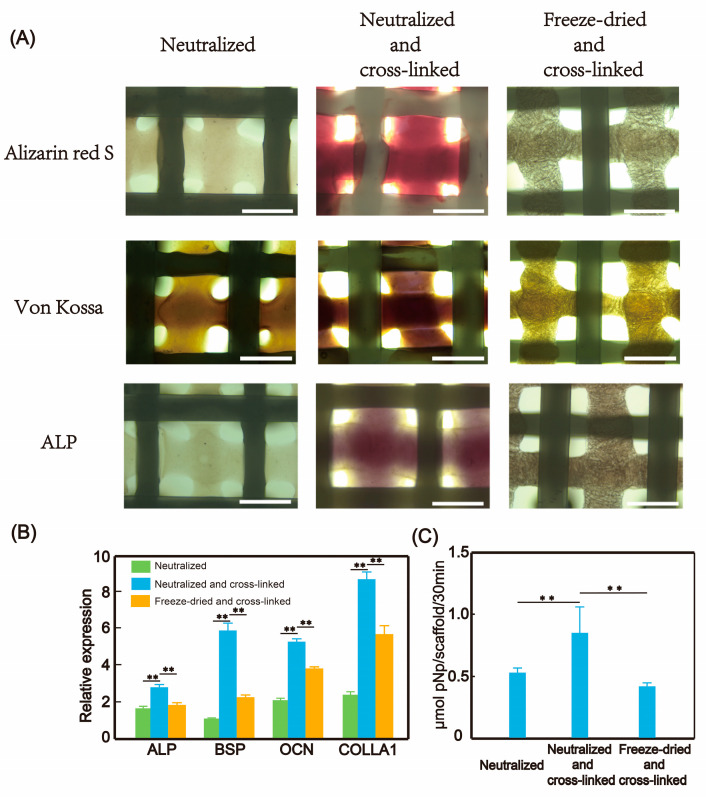
Differentiation of BMSCs after 21 days of culture in the hybrid scaffold with osteogenic medium. (**A**) Microscopic images of alizarin red S, von Kossa, and ALP staining in the hybrid scaffold with post-treatments, scale bar: 500 μm. (**B**) PCR analysis results of osteogenesis-related genes in the hybrid scaffold with different post-treatments, with the results of the control group (cells cultured under the same conditions but without the scaffold) set as 1, N = 3; data are means ± SD. ** *p* < 0.01. (**C**) ALP activity analysis results in the hybrid scaffold with different post-treatments, N = 3; data are means ± SD. ** *p* < 0.01.

We systematically assessed the osteogenic differentiation potential of bone marrow-derived mesenchymal stem cells (BMSCs) cultured on collagen hybrid scaffolds. First, the cells were seeded onto the cross-linked scaffolds and cultured in a normal medium for 7 days, after which osteogenic medium was added and the culture continued for 21 days. After the 21-day induction period, osteogenic differentiation was evaluated using various analytical methods, including alizarin red S staining, von Kossa staining, alkaline phosphatase (ALP) staining, and quantitative ALP activity assay. Calcium deposits were detected as dark red and black colors by alizarin red S and von Kossa staining, respectively (Figure 7A), confirming the functional expression of osteogenic differentiation. Moreover, cells in all three groups of scaffolds showed significantly enhanced ALP activity after induction. For ALP activity detection, we found that the neutralized and cross-linked group had the best effect (Figure 7C). Meanwhile, we performed PCR analysis on cells in the three groups of scaffolds to investigate the expression levels of osteogenesis-related genes, and the results showed similar trends (Figure 7B). The expression levels in all three groups of scaffolds were up-regulated, with the neutralized and cross-linked group having the highest expression, followed by the freeze-dried and cross-linked group and the neutralized group having the lowest expression. BMSCs can differentiate on different collagen surfaces. The preferential distribution of calcium deposits and ALP markers in collagen-rich areas indicates that scaffolds containing collagen may provide better osteogenic differentiation potential compared to pure PCL scaffolds.

## 4. Discussion

In recent years, the progress in the field of tissue engineering has increasingly highlighted the crucial role of scaffolds. However, the issue of how to optimally integrate mechanical properties with biocompatibility remains unresolved. From the perspective of developmental biology, tissues originate from the differentiation and proliferation of stem cells, with their mechanical properties transitioning from a soft to a hardened state. In contrast, in adult tissue regeneration through engineering strategies, the scaffold must match the mechanical properties of the surrounding tissue while providing immediate structural stability and having an appropriate porosity to support cell activity [21,22,23]. In most cases, the mechanical properties required for implantation differ from those needed for stem cell adhesion and differentiation. This approach essentially requires the development of composite materials, where one component ensures the stability of the implant, and the other promotes stem cell adhesion and differentiation. In this study, we chose a synthetic polymer to provide structural stability and collagen as the bioactive component.

Although low-temperature printing methods are widely used for the manufacturing of collagen scaffolds [24,25], they typically produce porous structures rather than nano-fiber structures that mimic the natural extracellular matrix. Extensive literature has demonstrated that nano-fiber structures significantly enhance biological functions [26,27], which is a major advantage of the collagen printing technology described in this paper. Adjusting the pH value to improve the printability of dialyzed collagen also avoids the issue of collagen fibers becoming too dense due to increased concentration, which can affect cell growth and proliferation. The transition of tissue-engineering scaffolds from research to clinical application is a lengthy process, mainly involving two key aspects: the selection of biomaterials and manufacturing methods. In this study, we used two clinically approved materials and developed a simple and practical manufacturing method. Avoiding the use of organic solvents, eliminating additional additives, and using clinical-grade materials together enhance the potential for clinical translation and accelerate the regulatory approval process.

Based on this method, the scaffold design can be further optimized to achieve mechanical properties and pore structures that are more suitable for specific tissue regeneration needs. In addition, before clinical transplantation, comprehensive and systematic experimental studies should be conducted, including using computer-aided design to accurately determine key scaffold parameters such as degradation kinetics, mechanical properties, and bioactive characteristics. This systematic approach will help optimize scaffold properties to meet specific clinical needs and ensure predictable in vivo performance.

## 5. Conclusions

In this paper, a versatile multi-materials printing method for hybrid scaffolds was developed, enabling the composite printing of collagen and thermoplastic elastomers at room temperature. The rheological properties and printability of pure collagen solutions were studied, and the impacts of various post-treatment methods on the bioactivity of collagen were explored, leading to the successful printing of collagen scaffolds with porous/nano-fiber morphologies of the extruded filaments. Through the development of PCL large-span printing technology, the hybrid printing of collagen and PCL was achieved. A continuous interconnected porous network was formed between the materials, which facilitated the exchange and permeation of nutrients and ensured uniform cell seeding and activity in subsequent long-term culture. The higher the proportion of collagen in the hybrid scaffold, the more cells were seeded. Depending on different tissue regeneration needs, the mechanical properties and cell seeding number of the hybrid scaffold could be adjusted by changing the ratio of collagen and synthetic polymer. The long-term three-dimensional culture of human fibroblasts on the hybrid scaffold showed that cells initially grew on the collagen fibers, and after about 10 days, cells were also able to cover the entire PCL fibers. BMSCs successfully achieved osteogenic differentiation on the hybrid scaffold, demonstrating the good biocompatibility of the scaffold. The culture of fibroblasts and BMSCs as test cells for soft tissue and hard tissue regeneration, respectively, revealed the great clinical application prospects of the collagen and synthetic polymer hybrid scaffold.

## Data Availability

Data are available from the corresponding author upon reasonable request.
